# The Built Environment and Active Travel: Evidence from Nanjing, China

**DOI:** 10.3390/ijerph13030301

**Published:** 2016-03-08

**Authors:** Jianxi Feng

**Affiliations:** Department of Urban Planning and Design, School of Architecture and Urban Planning, Nanjing University, Hankou Road 22, Nanjing 210093, China; jxfup@nju.edu.cn; Tel.: +86-25-8368-6002

**Keywords:** active travel, mobility, health, built environment, China

## Abstract

Background: An established relationship exists between the built environment and active travel. Nevertheless, the literature examining the impacts of different components of the built environment is limited. In addition, most existing studies are based on data from cities in the U.S. and Western Europe. The situation in Chinese cities remains largely unknown. Based on data from Nanjing, China, this study explicitly examines the influences of two components of the built environment—the neighborhood form and street form—on residents’ active travel. Methods: Binary logistic regression analyses examined the effects of the neighborhood form and street form on subsistence, maintenance and discretionary travel, respectively. For each travel purpose, three models are explored: a model with only socio-demographics, a model with variables of the neighborhood form and a complete model with all variables. Results: The model fit indicator, Nagelkerke’s ρ^2^, increased by 0.024 when neighborhood form variables are included and increased by 0.070 when street form variables are taken into account. A similar situation can be found in the models of maintenance activities and discretionary activities. Regarding specific variables, very limited significant impacts of the neighborhood form variables are observed, while almost all of the characteristics of the street form show significant influences on active transport. Conclusions: In Nanjing, street form factors have a more profound influence on active travel than neighborhood form factors. The focal point of the land use regulations and policy of local governments should shift from the neighborhood form to the street form to maximize the effects of policy interventions.

## 1. Introduction

Over the last three decades, physical inactivity has been widely recognized as a major public health challenge around the world. Solid epidemiologic evidence has confirmed that physical inactivity is an important risk factor for chronic diseases, including coronary heart disease, diabetes, strokes and psychological depression [1,2,3]. According to research from Cambridge, inactive lifestyles are responsible for about 676,000 deaths around the world each year [4]. A major activity of public health organizations has become the promotion of physical activity levels [5]. Active travel (*i.e.*, walking and cycling), as a major form of physical activity in people’s daily lives, is believed not only to help mitigate the adverse health effects of physically-inactive lifestyles, but also to curb the problems caused by auto-dependence, such as traffic congestion and air pollution, and therefore is highly recommended by scholars and policymakers as a mechanism that provides a variety of benefits to individuals, businesses and governments [1,2,6]. As a result, the research on how to encourage active travel has attracted increasing attention from health researchers, transport planners and geographers.

Various studies have demonstrated that active travel is influenced by both individual factors, such as socio-demographic characteristics and attitudes, and the built environment in which the individuals live and move [6,7]. Previous studies investigating the relationships between the built environment and active travel often emphasized more neighborhood features and the macro-scale of the physical environment, such as the famous 3Ds—density, diversity, design—summarized by Cervero and Kockelman [8], general accessibility and walking accessibility in particular [9]. Results differ across studies, but a recurrent finding is that the likelihood of use of active travel is higher in denser, more diversified urbanized settings [1,3]. The effects of accessibility to employment, shopping, leisure and transport facilities on active travel are also mixed, but often positive [8]. For instance, Bopp *et al.* found that public transport usage is positively associated with active commuting, which is most likely due to the fact that public transit users must walk or bicycle to access the transit line [10], while other scholar found no significant linkages between public transport accessibility and active commuting [1]. The findings appear least equivocal for access to green space, which usually has a positive impact on active travel, especially for walking [11].

Compared to the abundant studies on the relationship between neighborhood form and active travel, the influences of street forms and micro-scale features in individuals’ use and experience of neighborhood environments, such as the presence of illegal parking on sidewalks and building setbacks, are relatively less examined. Scholars have theoretically proposed the linkage between street form and active travel. For instance, Southworth proposed that six criteria for the design of a successful pedestrian network: connectivity; linkage with other modes; fine-grained land use patterns; safety; quality of path; and path context; the latter two criteria actually refer to the street form [12]. Nonetheless, the relationship between street environment and active travel is less empirically examined. Particularly, we do not know explicitly which aspects of built environments (*i.e.*, neighborhood form or street form) have more profound influences on people’s active travel choices. The limited evidence from Western countries seems to suggest that the neighborhood form is a more decisive factor that influences resident’s active travel, compared to street forms [1]. However, what is the situation in urban China? Do the findings based on Western cities also imply the Chinese settings?

Despite the known studies on the relationship between the built environment and active travel, the existing literature has drawn disproportionally on data from urban areas in Western countries [7,13]. Little is known about the situation of active transport and its determinants in the rest of the world, especially in China, where the social, economic and cultural contexts are quite different from the Western world. Additionally, Chinese cities also have different spatial structures and configurations than Western countries. The most notable characteristics are their high density and high levels of mixed land use [13]. Chinese cities generally have a very high residential density compared to Western cities. The average urban density in the six big cities (Beijing, Shanghai, Tianjin, Guangzhou, Hangzhou and Ningbo) is 146 persons per ha, which is approximately ten-times the density of cities in America, Australia and New Zealand (15 persons per hectare) and over 2.5-times the density of Western European cities (55 person per hectare) [14]. Moreover, the spatial quality of the built environment in different districts of a city could be very different. The newly-developed areas are generally equipped with separate bicycle lines and well-paved sidewalks, which are generally walking friendly. While in some areas of the old parts (e.g., the urban village), the aesthetic quality and safety of the built environment could be quite low [15]. The streets are crowded, and in most situations, the sidewalks, if there are any, are blocked by illegal parking. There could also be informal employees working as peddlers and vendors in the streets [16]. Though there are also differences in the quality of the built environment in Western cities, the differences in Chinese cities are generally much greater [16]. Apparently, these special attributes of the built environment in Chinese cities will have profound implications on residents’ travel choices and make the Chinese context a useful case in which to examine how the built environment influences people’s travel behavior in general and active travel in particular.

Therefore, there are two related research questions: (1) What is the general situation of active travel among the urban residents in China? (2) How do the attributes of the built environment influence active travel, and in particular, are the direction and the extent of the influence of the neighborhood form and the street form on active travel choices the same? Specifically, regarding the influence of built environments, we propose two hypotheses:

1. Both the neighborhood form and the street form tend to have significant impacts on active travel;
2. Compared to the neighborhood form, the street form tends to have more profound influences on active travel, given the larger variances in street form in Chinese cities.

For our analysis, we use data from the 2012 Nanjing Residents Travel Survey (NRTS). We answer these research questions in the following five sections. Section 2 starts with an overview of the data, measures of the built environment and the methods used. In Section 3, we turn our attention to a descriptive analysis of our dataset and a number of regression models. The results of this analysis will be further discussed in Section 4. Finally, conclusions are drawn in Section 5.

## 2. Materials and Methods

To examine the link between the built environment and active travel, results from a survey of residents’ travel behavior in Nanjing, China, were used. The following section details the general situation of the study area, the data collection effort, the measures of the built environment and the demographic covariates included in the analyses.

### 2.1. Study Area and the Data

**Figure 1:**
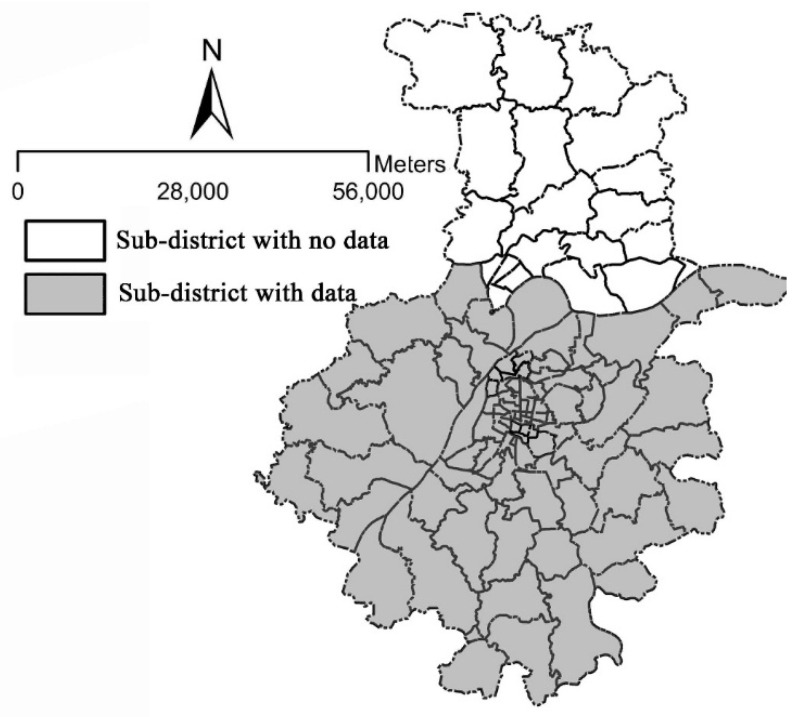
Map of Nanjing in China.

We studied our hypotheses in Nanjing, China. This city, the capital of Jiangsu Province, is the second largest commercial center in East China. It covers an area of 4723 square kilometers and had a total population of 6.38 million in 2012 [17]. We conducted the quantitative analysis using data from the Nanjing Residents Travel Survey, which was conducted by the Nanjing Urban and Transportation Planning and Designing Institution Limit Cooperation. The survey was sponsored by the Nanjing Transportation Planning Committee and was used for “The Annual Transportation Report of Nanjing 2012”. The survey covered 10 districts of Nanjing, and the dataset includes about 6000 respondents of 2000 households (Figure 1).

The sub-sample size of each district is proportional to its population. More traffic analysis zones (TAZs) are selected in the districts with large populations. In each TAZ, 150 respondents from approximately 50 households were selected using a systematic random sampling method. The sampling frame was the list of households registered in the sub-district. This travel database includes the socioeconomic and demographic attributes of the respondents, such as gender, age, education level, occupation, household income, household car ownership and detailed information, derived from one-day travel diaries completed on Tuesdays, Wednesdays and Thursdays in December 2012 and January 2013. It is notable that though the survey months are not a typical time for people to walk or cycle, such that some of the respondents might choose other modes than active travel, because of cold weather, the investigation of the influences of built environments on active travel based on these data still has scientific and practical relevance.

### 2.2. Built Environment Measures

According to the behavioral model of the environment [3,18], which provides a theoretical and conceptual framework for the way in which the built environment relates to people’s behavior in general and travel behavior in particular, three environmental components are proposed: the origins and destinations of trips, the route characteristics of trips and the characteristics of the area around origins and destinations. The first component refers to the types (e.g., home, working places, schools) and the locations of origins and destinations. Route characteristics of trips include spatio-physical aspects, such as the distance between the origin and destination or the design of the roadway, and spatio-behavioral aspects, such as the volumes of cars, bicycles or people on the roadway. The characteristics of the area around the origins and destinations are the spatio-physical aspects of the environment, such as the types and the intensity of the uses of land (as proxies for activities that take place and their intensity). In this paper, three components are also developed to present built environments. For the first component, we distinguished three different travel purposes (subsistence, maintenance and discretionary), which to some extent could represent the types of destination locations. In this study, subsistence activities include work, work-related activities and school activities. Maintenance activities refer mainly to shopping activities and seeing a doctor, while discretionary activities include sports, tours, entertainment and social activities.

The second and third components are referred to as the street form and neighborhood form, respectively, in this study. Inspired by the seminal work of Cervero and Kockelman [8], for each component, the three dimensions—density, diversity and design—of the built environment are investigated. Table 1 shows the detailed indicators for each dimension. It is notable that the analysis unit of the indicator is a circle whose center is the TAZ centroid and radius is 1000 m, which is generally used as the threshold distance for walking and cycling [9] (except the distance to the nearest open/green space). For instance, population density is calculated as the ratio of the population and area of this circle. The ”percentage of the street with separate bike lanes” refers to the ratio of the number of separate bike lanes and the total number of streets within the circle. Land use mix simultaneously accounts for the variety and prevalence of different functions in the area. Following Cervero and Kockelman [8], we calculated an entropy index:

$$s=-\sum_{j}\frac{\left[P_{jk}\times \ln(P_{jk})\right]}{\ln(J)}$$

In this equation, *S* refers to land use mix (entropy), *j* is the type of land use (*j* = 1, 2, …,J); *k* the TAZ of Nanjing city; *P_jk_* the proportion of land use *j* within the TAZ. The entropy ranges from 0 (homogeneity-only, one type of land use) to 1 (heterogeneity, shares of uses evenly distributed over all land use categories). We include six land use types with the highest relevance for residents’ daily activities: residential, commercial, public, industrial, offices and research sites and parks and recreational use.

### 2.3. Demographic Covariates

To explicitly examine the impacts of the built environment on active travel, the current study controlled the individual socio-demographics in the regression model. The existing research shows that women are more likely to use active travel than men [7,19]. Age is a significant factor influencing travel behavior. Young adults are less inclined to use non-motorized transport than older people [20]. The influence of the level of education and household on mode choice is inconsistent. Some authors conclude that high educational achievement is positively related to the use of active transport, while others have found the reverse effects [19,21]. Regarding the influence of household income, Cao *et al.* (2006) has observed a positive influence on the number of car trips [22]. However, others find no significant relationship between household income and mode choice [23,24]. Household size is generally positively related to less active transport use, and the presence of children tends to increase the use of cars and decrease the use of active transport [21]. The effect of car ownership on mode choice seems more consistent. Higher levels of car ownership result in increased car trips and diminishing trips through other transport modes [25]. Therefore, in this study, gender, age, educational level, household income, household size, the presence of a pre-educated child and household car ownership were included in the analyses as covariates.

## 3. Analysis and Results

### 3.1. General Situations of Active Travel in Nanjing

Basic descriptive statistics and frequencies were used to describe the sample (Table 2 and Table 3). Different from Western countries, especially in the U.S., where private car use makes up more than 80% of all out-of-home trips [26], active transport is still the dominant transport mode in Nanjing, which accounts for about 60% of total trips. Two interrelated reasons might explain this. First, car ownership rates in China, though fast growing, are still lower than the rates in most Western countries. According to the World Bank, in 2011, car ownership was 83 per thousand in China and 809 per thousand in the U.S. In 2008, car ownership was 544 per thousand in Germany and 37 per thousand in China [7]. Second, the differences in the built environment between Western countries and China might also be a reason. Muller’s (1995) four-stage model of intra-metropolitan transport and urban growth assumes that the dominant transport system in a certain era shapes the spatial structure of the city in the U.S [27]. In a similar vein, the land use pattern in most Chinese cities is developed and has accommodated non-motorized transport modes. The land use pattern, characterized with high density and land use mix, offers residents more opportunities to travel short distances than their Western counterparts who live in built environments shaped by fast transport modes. Therefore, walking and cycling are more prevalent in Chinese cities.

There are also considerable differences in the transport mode use for different purposes. For subsistence activities, the use of active transport is the lowest, and private car usage is the highest, while for maintenance activities, active transport makes up more than 70% of total trips. The difference actually manifests the importance and temporal-spatial rigidity of various activities and the consequential strategy people utilize to access these spatial opportunities. Given the importance of subsistence activities, people generally search for employment in the entire city, and therefore, the travel distance for employment is longer than for maintenance activities, which usually can be found locally. The differences in the temporal-spatial fixity of different activities are also important reasons for the variance in transport mode usage. Obviously, the differences in transport mode use necessitate separate analyses of the influences of the built environment on mode choices for different travel purposes.

### 3.2. Binary Logistic Regression Models for Subsistence, Maintenance and Discretionary Travel

Binary logistic regression analyses were conducted to examine the effect of the built environment on active travel choices for subsistence, maintenance and discretionary travel purposes, respectively (Table 4, Table 5 and Table 6). Since the whole data are hierarchical, such that the survey data and demographic attributes are at the individual level while the data of built environments are at a higher level (TAZ), multilevel regression modelling might be more suitable for the analysis. However, due to the limited number of TAZs surveyed in the data, it cannot meet the model requirement for the least numbers of units of a high level (as a rule of thumb: there should be at least 30). We still use binary logistic regression, but keep in mind that the results are to some extent biased. Whether or not to use active travel (*i.e.*, public transport and private cars are converged together as the not taking active travel category) were used as dependent variables. Given that the idea of the study is to examine which built environment attributes have more profound influences on walking and cycling, three models are explored: a model with only socio-demographics (Model A), a model with variables of the neighborhood form (Model B) and a complete model with all variables (Model C). The pseudo R-square statistic, Nagelkerke’s ρ^2^, indicates the percentage of the variance of the dependent variable that can be explained by the independent variables [7] and “Sig.” in the table refers to significance.

It is worth noting that we checked the multicollinearity properties of the explanatory variables to avoid spurious and erroneous modeling. For that purpose, the variance inflation factor (VIF) was used. All of the selected variables had VIF values between 1.0 and 2.0, except for the “percentage of bike lanes”, for which VIFs ranged between 2.37 and 2.58, depending on the model specification. A commonly-accepted cutoff point signaling multicollinearity problems in logistic regression is 2.5 [27], so we decided not to exclude any selected variables in the modeling to maintain consistency across the models.

As shown in Table 4, Table 5 and Table 6, Nagelkerke’s ρ^2^ of models for subsistence travel is 0.115 (Model A), 0.139 (Model B) and 0.209 (Model C). Nagelkerke’s ρ^2^ of models for maintenance travel is 0.079 (Model A), 0.134 (Model B) and 0.207 (Model C). Nagelkerke’s ρ^2^ of models for discretionary travel is 0.075 (Model A), 0.129 (Model B) and 0.173 (Model C). Apparently, the overall model fit is lowest for discretionary trips. This might be because leisure trips are more determined by other personal preferences and values, which are not included in the model [28].

However, when calculating the contribution of the built environment for different travel purposes, it is found that for subsistence travel, the contribution of the built environment is the lowest (the contribution of the built environment for subsistence activities is (0.209 − 0.115)/0.209 = 45%; the contribution for maintenance activities is (0.207 − 0.079)/0.207 = 62%; the contribution of the built environment for discretionary activities is (0.173 − 0.075)/0.173 = 0.57%). People usually choose employment opportunities from all over the metropolitan area, while when people make shopping or leisure trips, they are more inclined to use local facilities. Since all of our built environment variables are residential area-based, the influence of these local built environment indicators are therefore more pronounced for maintenance and discretionary activities and less influential in determining the mode choice for subsistence activities.

Nagelkerke’s ρ^2^ also indicates that the influence of the neighborhood form is not as strong as the street form on active travel choices. For subsistence activities, Nagelkerke’s ρ^2^ increases by 0.024 when neighborhood form variables are included and increases by 0.070 when street form variables are taken into account, suggesting that street form variables can explain more variance of the dependent variable than the neighborhood form. A similar situation can be found in the models of maintenance activities and discretionary activities.

Regarding specific variables, very limited significant impacts of the neighborhood form are observed while almost all of the influences of the street form are statistically significant. Population density shows significant influences on the active travel choice in Model B of subsistence and discretionary activities. In line with previous studies [29], the higher the density is, the more likely that people will choose active travel. However, when street form variables are controlled, the impacts become insignificant. Surprisingly, the land use mixture shows no significant influences in all models, which is in contrast with the findings from Western countries. The design dimension of the neighborhood form—distance to the nearest green/open space—demonstrated profound influence on active travel. In the six models, it exerts significant influences, even when the characteristics of street form are controlled. Given that open/green spaces, especially medium- and small-sized parks, are far less prevalent in most Chinese cities than in Western countries [15], good accessibility to open/green spaces could considerably encourage the use of active transport.

Almost all of the characteristics of the street form show significant influences on active transport. The density of street crossings is positively related to active travel. The areas with a high density of street crossings are generally the areas constructed at a walkable human scale [30]. In addition, more street crossings mean more blocks, which makes driving cars inconvenient. The diversity factor of the street form—the percentage of roads with separate bike lanes and the percentage of roads with sidewalks—also exerts a significant impact on active travel choice. Similar to the findings from Western cities, the more numerous the separate bike lanes and sidewalks, the higher propensity for the residents to use active transport [3]. In addition to the direct effects that the infrastructure has on making active travel more convenient and fluid, the indirect effects that they have on making travelers feel safe cannot be ignored. The findings highlight the importance of the pedestrian/cycling infrastructure in promoting active transport. Regarding the influence of the design aspects of the street form, the analysis results of all three models demonstrate consistently negative relationships between illegal parking on the street and the use of active travel, suggesting that having to traverse walking/cycling environments without major barriers is a crucial factor for promoting active travel.

Unlike the findings in Western cities [2,5], building setbacks seem positively related to active travel in our analysis, except for subsistence travel. Chinese cities are characterized by high density with intensely constructed buildings, so that the setbacks are usually much smaller than in Western cities. The buildings in China make the streets crowded and not as easy to walk through. Large building setbacks make more spaces for walking and cycling and, therefore, are positively related active travel. However, too large a building setback, generally related to less diversified and tedious streetscapes, may have negative influences on walking/cycling, as confirmed from Western evidence. In other words, the relationship between building setbacks and active travel may be quadratic rather than linear. The different effect of building setbacks on active travel could also result from potential confounding factors, such as the average width of sidewalks/bike lanes and streets and the volume of traffic, which are not included in this analysis.

In addition to built environment factors, individual socio-demographics also exert notable influences on active travel. Compared to men, women are more likely to use active transportation for subsistence travel. The limited car access of women and short commuting distance might account for the differences. However, for discretionary travel, the influence is insignificant. One possible reason might be that women and men tend to have more joint recreational activities, and therefore, the differences in mode choice are minor. Given the special divisions of household tasks in Chinese households caused by special cultural norms and ideology, which will influence the related travel-activity patterns between male and female heads, the gendered differentials in active travel deserve further research. Generally, age is positively related to active transport, though some of the effects are not significant. People with high educational levels seem more inclined to use motorized transport than active transport, especially for subsistence travel. Highly-educated people tend to have more professional jobs and special requirements for goods and recreational facilities that cannot be found readily in the proximity of home. The influence of income is rather moderate, which is probably caused by the relationship with the educational level (a high education level is related to high income level). Household size only has significant effects on active travel choice for subsistence activities when street form variables are not controlled. The presence of a pre-educated child tends to reduce the propensity to use active travel for recreational activities. For the sake of convenience and safety, this result makes intuitive sense. Household car ownership, as expected, is highly negatively related to active travel for all travel purposes.

## 4. Discussion

Previous studies using data from Western cities have found consistent associations between walking for transportation purposes and density, land use mix and other neighborhood form factors, such as the proximity of non-residential destinations. In contrast, empirical evidence has less consistently found a relationship between transportation, walking and pedestrian infrastructure, such as sidewalk presence and conditions. It seems that in Western countries, compared to street forms, the neighborhood form is a more decisive factor that influences resident’s active travel. However, in China, the reverse is observed. As indicated by Nagelkerke’s ρ^2^, street form factors are more powerful in explaining the variance of the dependent variable than neighborhood form factors for all three travel purposes. The performance of specific built environment variables also shows the same trends. Density and land use mix, which are considered the two most important factors influencing the travel behavior of residents [31], show rather limited influences on active travel. Especially, when the variables of street form are controlled, the influences become negligible. In contrast, all of the street form factors are consistently correlated to active travel in the current analysis.

While there might be several reasons for different effects, the different spatial structures of urban spaces caused by different socio-cultural backgrounds between Chinese cities and Western cities could be an important one that cannot be ignored. In Western cities, the differences in the neighborhood form in different districts are generally larger than the differences in the street form in terms of the pedestrian/cycling infrastructure [15]. In the center of the city, the density could be very high and there could be a high level of land use mix, while in the suburban area, the density is usually rather low, and a single land use is overserved. However, the differences in the walking environment are not as big as the density and land use mix in that they are generally not so crowded and built with aesthetic views. However, in China, the differences in the neighborhood form in terms of density and land use mix are not as notable as the differences in the street form [16]. Due to land ownership, the municipal government still fundamentally determines land allocation, transportation investment and operation, as well as the provision of commercial and social services. Through making and implementing detailed land use plans, exercising charges on users of urban land, infrastructure and other facilities, the government maintains the intensified use of urban land.

Moreover, urban China has a tradition of mixed land use. In the pre-reform era (from 1949 to early 1980), urban settlement was dominated by the *Danwei* system in various aspects [32]. *Danwei* was the basic unit of urban China before reform. It provided its employees a “comprehensive package of welfare and service” in addition to salaries, including housing, daily life facilities, such as dining halls, bathing houses, shops, education, recreation and medical care facilities, like kindergartens, clinics and sports fields [33]. These facilities and housing are usually built along with their production activities to make the lives of its employees more convenient and thus promote the efficiency of their productivity. Although the land use and housing reform introduced in the early 1980s has transformed *Danwei* from a multi-functional work unit to one that basically provides economic rewards to its employees for their work, there are other national planning guidelines guaranteeing land use in cities maintained with a certain degree of mixture. For example, the “Codes for Urban Residential Area Planning and Design”, which was enacted on 1 February 1994 by the Ministry of Constructions, China, specifies basic provisions of education, commercial and other public service for residential projects of a deferent level, regardless of whether they were developed by private developers or by SOEs (State-Owned Entrepreneurs) [7]. In other words, these guidelines in China actually led to a mixture of different land uses in cities.

However, the differences in the quality of urban space in general and walking environments, in particular in a transitional era of urban China, are considerable. China has been experiencing unprecedentedly fast urban expansion and transformation, which have resulted in huge disparities in the quality of urban spaces in the same city, even sometimes in the same district [16]. The disparities in the quality of the urban space are manifested in several areas, including in the provision of public facilities, open and green space, the aesthetic quality and safety, among which, the differences in walking and cycling infrastructure in different areas are also considerable. The differences in old and newly-developed areas are also distinguished. In the old parts of the city, it is quite common to have narrow streets with no separate sidewalks/bike lanes and to be crowded with informal peddlers [15]. The newly-developed areas are generally built with good pedestrian/cycling infrastructure and green trees along streets to provide shade in hot weather and other amenities. The diverse group compositions in street forms to some extent amplify the impact on active travel.

Obviously, the special development history, institutional arrangements and development stages, interacting with socio-economic contexts, make the structure and distribution of land use. As such, the form, sizing and scaling of urban plots and the sizing and scaling of street networks of Chinese cities are all different from their Western counterparts. The special spatial forms then shape the behavior in general and active travel in particular of people who are embedded in and interacting with the built environments at varying spatial scales, as indicated above.

## 5. Conclusions

Active travel as “green” transport not only reduces congestion, has low environmental impact and conserves energy without producing air and noise pollution, but it also has the potential value of promoting individual health conditions and enhancing community coherence and other social benefits. Over the last three decades, extensive literature has investigated the way and extent that the built environment influences active travel. However, most of the empirical evidence is based on the data from cities in the U.S. and Western Europe. The research examining the situation in China is limited. This study, to our knowledge, is one of the first efforts aimed at exploring and comparing how the different components of the built environment—the neighborhood form and street form—influence active travel. The paper, therefore, sets out to answer the two research questions: (1) What is the general situation of active travel among the urban residents in China? (2) What are the influences of the neighborhood form and the street form on active travel in the special spatial forms of urban China?

In answering the first question, we found that active travel is still a dominant transport mode in Nanjing in 2012. It makes up about 60% of total trips, and the percentage for maintenance activities is even higher. Relatively lower income levels and car ownership, as well as the compact pattern of the built environment, which is constructed and accommodated to non-motorized transport, are the underlying reasons. However, we might keep in mind that China currently is experiencing very fast economic growth with more than an increase of 8% GDP per year on average. Fast economic growth has resulted in individual affluence, and the car ownership of urban residents has rapidly grown as well from 3.4% to 18.6% between 2005 and 2011 [34]. Chinese cities, therefore, run high risks of spreading over larger areas, resulting in lower densities, a development comparable to previous processes in Western countries. As a result, the proportion of active travel will also decrease dramatically. Land use policy and transport policies that guide Chinese cities towards walkability and environmental sustainability seem to have become one of the biggest challenges for policymakers in urban China.

For the second question, we found that the street form tends to exert more profound influences on active travel than the neighborhood form. This result is different from the empirical evidence of Western cities. The character of urban China might influence the result because urban areas have large differences in the quality of urban spaces in general and the walking environment in particular, but relatively small differences in density and land use mix. Obviously, socio-economic backgrounds, institutional contexts and the development stage of China, which caused the special spatial characteristics of Chinese cities, matter in the research on active travel, its determinants and health and transport research. More theoretical and empirical research of Chinese cities could expand the existing literature and provide a deeper, more complete understanding of the relationship between active travel and the built environment and are therefore a high priority for future research.

The findings of this study may have important implications for planning practices. While current land use regulations and policies have emphasized land use mixture and public facility provisions at the neighborhood level, relatively little attention has been paid to the street form in terms of the provisions of pedestrian/cycling infrastructure, the traversability of the street and the aesthetic quality of the walking environment. However, the analyses in this study suggest that the characteristics of the street form are even more important than the neighborhood form in shaping residents’ mode choices in urban China. Therefore, it seems that the focal point of the land use regulations and policy should shift from the neighborhood form to the street form. Efficient provisions of separate bike lanes and sidewalks, green trees along sidewalks to provide shade in hot weather and other amenities, such as seats for rest and a well-managed parking system to remove illegal parking and make the street traversable, should be placed at the core of policy interventions. In addition, the government should also encourage the neighborhood to be constructed at the human scale of small blocks with dense street networks, to provide more open/green spaces around the residential area. At any rate, since the Chinese government is entering into an era of having relatively limited financial and land resources compared to the two decades before, a more targeted package of land use policies could help maximize the effects of policy interventions.

It should be noted that the current work has some limitations. One obvious limitation is that the study uses cross-sectional data and binary logistic regression models, which can only demonstrate the correlation, not the causality, between built environments and active travel. Better approaches in terms of dataset design and statistical models that offer deeper examination of the direct and indirect relationships, interactions and hypothesized paths of causality between the environment and behavior are promising areas for future research. Second, the paper includes only objective measures of the built environment. Scholars have emphasized the importance and necessity of including subjective measures like the perceptions of the street environment in the study of built environments and active travel [35]. Therefore, both perceived and objective measures should be included in future research.

## Figures and Tables

**Table 1:** Indicators for the built environment.

	Neighborhood Form	Street Form
Density	Population density	Density of street crossings
Diversity	Land use mixture	Percentage of streets with a separate bike lane
		Percentage of streets with sidewalks
Design	Distance to nearest green/open space	Percentage of streets with illegal parking
		Building setback

**Table 2:** Profile of the sample.

Variables	Categories	Frequency (Mean)	Percentage
Gender	Male	2441	48.3
	Female	2610	51.7
Age	20 to 40	2070	41.0
	40 to 60	2372	47.0
	More than 60	609	12.1
Education	Middle school and below	774	15.3
	High school	1776	35.2
	College and above	2501	49.5
Household income	Less than 50,000	3296	65.3
	50,000 to 100,000	466	9.2
	More than 100,000	1289	25.5
Car ownership	No car	3720	73.6
	Car ownership	1331	26.4
Household size		3.04	100

**Table 3:** Mode choice for different travel purposes.

Travel Purposes	Active Travel	Public Transport	Private Car	Total
Subsistence	2299 (54.6%)	943 (22.4%)	967 (23.0%)	4209 (100%)
Maintenance	1352 (71.8%)	304 (16.1%)	228 (12.1%)	1884 (100%)
Discretionary	471 (64.8%)	207 (28.5%)	49 (6.7%)	727 (100%)
Total	4122 (60.4%)	1454 (21.3%)	1244 (18.2%)	6820 (100%)

**Table 4:** Binary logistic model for subsistence travel.

Variables	B	Sig.	Odds Ratio	B	Sig.	Odds Ratio	B	Sig.	Odds Ratio
*Socio-demographics*									
Gender (male = ref.)									
Female	0.877	0.000	2.404	0.882	0.000	2.417	0.642	0.000	1.901
Age (more than 60 = ref.)									
20 to 40	−0.605	0.035	0.546	−0.625	0.030	0.535	−0.318	0.276	0.728
40 to 60	0.044	0.876	1.045	−0.042	0.883	0.959	0.221	0.442	1.247
Education (middle school and below = ref.)									
High school	−0.465	0.002	0.628	−0.517	0.001	0.596	−0.352	0.024	0.703
College above	−0.855	0.000	0.425	−1.003	0.000	0.367	−0.663	0.000	0.515
Income (less than 50,000 = ref.)									
50,000 to 100,000	0.610	0.000	1.840	0.331	0.014	1.392	0.047	0.744	1.048
More than 100,000	0.595	0.000	1.813	0.618	0.000	1.856	0.250	0.145	1.284
Household size	−0.073	0.526	0.929	−0.159	0.178	0.853	−0.248	0.042	0.781
Pre-education Child (no = ref.)	0.103	0.671	1.108	−0.118	0.632	0.889	0.229	0.368	1.258
Car ownership (no car = ref.)	−0.578	0.000	0.561	−0.398	0.008	0.672	−0.256	0.109	0.774
*Built Environment (Neighborhood form)*									
Population density				0.058	0.034	1.060	0.012	0.716	1.012
Land use mixture				0.073	0.221	1.075	0.064	0.906	1.066
Distance to the nearest green/open space				−0.028	0.000	0.973	−0.023	0.000	0.977
*Built Environment (Street form)*									
Density of street crossing							0.141	0.004	1.151
Percentage of separate bike lanes							0.556	0.000	1.744
Percentage of sidewalks							0.153	0.002	1.165
Illegal parking on sidewalks							−0.938	0.000	0.391
Building setback							0.068	0.129	1.070
Constant	0.887	0.053	2.428	1.477	0.003	4.378	1.528	0.005	4.607
Nagelkerke’s ρ^2^			0.115			0.139			0.209
No. of Cases			4204			4204			4204

**Table 5:** Binary logistic model for maintenance travel.

Variables	B	Sig.	Odds Ratio	B	Sig.	Odds Ratio	B	Sig.	Odds Ratio
*Socio-demographics*									
Gender (male = ref.)									
Female	0.644	0.000	1.903	0.605	0.000	1.830	0.333	0.006	1.395
Age (more than 60 = ref.)									
20 to 40	−0.376	0.019	0.686	−0.199	0.231	0.819	0.408	0.028	1.504
40 to 60	0.134	0.330	1.143	0.218	0.123	1.243	0.539	0.000	1.714
Education (middle school and below = ref.)									
High school	−0.120	0.396	0.887	−0.228	0.116	0.796	−0.144	0.339	0.866
College above	−0.594	0.000	0.552	−0.798	0.000	0.450	−0.458	0.009	0.632
Income (less than 50,000 = ref.)									
50,000 to 100,000	0.253	0.267	1.288	−0.301	0.218	0.740	−0.478	0.061	0.620
More than 100,000	0.783	0.003	2.189	0.787	0.011	2.196	0.778	0.024	2.177
Household size	0.029	0.859	1.029	−0.053	0.773	0.949	−0.056	0.762	0.946
Pre-education Child (no = ref.)	0.427	0.312	1.533	−0.131	0.765	0.877	−0.201	0.661	0.818
Car ownership (no car = ref.)	−0.656	0.012	0.519	−0.261	0.388	0.770	0.036	0.909	1.037
*Built Environment (Neighborhood form)*									
Population density				0.002	0.604	1.002	0.001	0.511	1.001
Land use mixture				0.077	0.407	1.081	0.043	0.665	1.044
Distance to the nearest green/open space				−0.054	0.000	0.948	−0.030	0.002	0.970
*Built Environment (Street form)*									
Density of street crossing							0.459	0.003	1.582
Percentage of separate bike lanes							0.209	0.064	1.232
Percentage of sidewalks							0.682	0.007	1.978
Illegal parking on sidewalks							−1.317	0.000	0.268
Building setback							0.732	0.000	2.079
Constant	0.701	0.171	2.016	1.644	0.010	5.174	3.206	0.000	24.680
Nagelkerke’s ρ^2^			0.079			0.134			0.207
No. of Cases			1884			1884			1884

**Table 6:** Binary logistic model for discretionary travel.

Variables	B	Sig.	Odds Ratio	B	Sig.	Odds Ratio	B	Sig.	Odds Ratio
*Socio-demographics*									
Gender (male = ref.)									
Female	−0.133	0.420	0.876	−0.134	0.427	0.875	−0.219	0.219	0.803
Age (more than 60 = ref.)									
20 to 40	−0.562	0.040	0.570	−0.293	0.302	0.746	−0.073	0.810	0.930
40 to 60	0.318	0.095	1.374	0.412	0.036	1.509	0.462	0.026	1.587
Education (middle school and below = ref.)									
High school	−0.366	0.067	0.694	−0.396	0.054	0.673	−0.274	0.196	0.760
College above	−0.358	0.156	0.699	−0.521	0.048	0.594	−0.156	0.587	0.856
Income (less than 50,000 = ref.)									
50,000 to 100,000	0.296	0.387	1.344	−0.090	0.802	0.913	−0.048	0.900	0.953
More than 100,000	0.835	0.031	2.304	0.878	0.032	2.405	0.739	0.067	2.095
Household size	0.169	0.425	1.184	0.188	0.402	1.207	0.153	0.484	1.166
Pre-education Child (no = ref.)	−1.652	0.006	0.192	−2.031	0.001	0.131	−2.344	0.000	0.096
Car ownership (no car = ref.)	−0.110	0.769	0.896	−0.070	0.859	0.933	−0.500	0.227	0.606
*Built Environment (Neighborhood form)*									
Population density				−0.019	0.002	0.981	−0.017	0.028	0.983
Land use mixture				−0.132	0.342	0.876	−0.128	0.374	0.880
Distance to the nearest green/open space				−0.054	0.000	0.947	−0.064	0.000	0.938
*Built Environment (Street form)*									
Density of street crossing							0.527	0.044	1.694
Percentage of separate bike lanes							0.600	0.003	1.822
Percentage of sidewalks							0.874	0.008	2.396
Illegal parking on sidewalks							−0.615	0.010	0.541
Building setback							0.430	0.048	1.537
Constant	0.260	0.693	1.298	1.601	0.041	4.956	0.949	0.312	2.583
Nagelkerke’s ρ^2^			0.075			0.129			0.173
No. of Cases			727			727			727
